# Land Subsidence in the Loess Plateau: SBAS-InSAR Analysis of Yan’an New District During 2017–2022

**DOI:** 10.3390/s25206298

**Published:** 2025-10-11

**Authors:** Yang Hong, Peng Chen, Yibin Yao, Liangcai Qiu, Hang Liu, Chengchang Zhu, Jierui Lu

**Affiliations:** 1 College of Geomatics, Xi’an University of Science and Technology, Xi’an 710054, China; 23210226091@stu.xust.edu.cn (Y.H.); hangliu@xust.edu.cn (H.L.); 19410070121@stu.xust.edu.cn (C.Z.); 22210061027@stu.xust.edu.cn (J.L.); 2 School of Geodesy and Geomatics, Wuhan University, Wuhan 430079, China; ybyao@sgg.whu.edu.cn (Y.Y.); liangcaiqiu@whu.edu.cn (L.Q.); 3 Key Laboratory of Geospace Environment and Geodesy, Ministry of Education, Wuhan University, Wuhan 430079, China; 4 State Key Laboratory of Geodesy and Earth’s Dynamics, Innovation Academy for Precision Measurement Science and Technology, Chinese Academy of Sciences, Wuhan 430077, China

**Keywords:** Yan’an New District, land subsidence, SBAS-InSAR, groundwater storage, fill thickness

## Abstract

Located on the Loess Plateau, the Yan’an New District (YND) has experienced significant geological instability due to large-scale mountain excavation and city construction (MECC). This study applied the Small Baseline Subset Interferometric Synthetic Aperture Radar (SBAS-InSAR) technique to 66 ascending Sentinel-1A SAR images acquired between January 2017 and May 2022 to investigate ground deformation patterns and influencing factors. Results show that the maximum subsidence rate reached −86 mm/year, with a maximum cumulative deformation of 400 mm. Groundwater storage was identified as the key natural driver, exhibiting a significant positive correlation (*r* = 0.4–0.8) with cumulative deformation with a two-month lag. Fill thickness emerged as the dominant anthropogenic factor, controlling the duration of soil consolidation and thus the deformation rate. Regulating groundwater extraction and improving recharge can effectively reduce subsidence risks. These findings provide scientific guidance for geological hazard early warning and urban planning in YND.

## 1. Introduction

Land subsidence is a geological and environmental phenomenon characterized by a regional lowering of the ground surface due to the compression and consolidation of loose surface strata, driven by both anthropogenic and natural factors [1]. With the acceleration of urbanization and the intensification of human activities, land subsidence has become a widespread and severe environmental and geological hazard. Notable examples have been reported in cities such as Beijing [2], Mexico City [3], and various regions in Italy [4]. This phenomenon poses significant threats to urban infrastructure, sustainable socio-economic development, and the well-being of affected communities. Therefore, accurately monitoring the spatiotemporal dynamics of land subsidence is essential for understanding its mechanisms and for providing a scientific foundation for disaster prevention and mitigation.

Currently, land subsidence monitoring primarily relies on discrete geodetic techniques such as leveling, Global Navigation Satellite Systems (GNSS), and triangulated elevation measurements. Conventional methods have the advantages of high single-point accuracy and strong reliability, but their sampling density is limited, making it difficult to achieve large-scale surface deformation monitoring. Moreover, they suffer from slow speed and high costs when applied to wide-area monitoring [5,6]. In contrast, Interferometric Synthetic Aperture Radar (InSAR), which utilizes synthetic aperture radar (SAR) data, offers several advantages over conventional methods. These include continuous data acquisition, all-weather operability, low cost, high precision, fine spatial resolution, and wide-area coverage [7,8]. InSAR has been widely applied in fields such as seismic deformation monitoring [9,10], urban subsidence detection [11], landslide monitoring [12,13], glacier tracking [14], and volcanic hazard assessment [15]. Among InSAR techniques, Differential InSAR (D-InSAR) extracts surface deformation by comparing multiple SAR images of the same region acquired at different times, achieving centimeter-level accuracy. Permanent Scatterer InSAR (PS-InSAR) measures land subsidence or deformation by analyzing the phase changes in stable permanent scatterers on the ground in a series of SAR images [16]. Permanent scatterers are often associated with man-made structures such as building roofs or bridges, which in many cases can provide stable radar reflections and support high-precision deformation measurements. However, recent studies have shown that structures such as bridges and large-span roofs are highly sensitive to temperature variations, and their daily thermal expansions and contractions may exceed one-quarter of the radar wavelength. This effect can compromise interferometric coherence and reduce the reliability of displacement measurements [17]. Therefore, when using such structures as permanent scatterers, the compatibility between the radar wavelength and the expected structural deformations should be carefully evaluated in advance. However, PS-InSAR technique may encounter decorrelation issues when performing long-time series monitoring due to the selection of a reference image. The Small Baseline Subset InSAR (SBAS-InSAR) technique further enhances performance by optimizing the selection of image pairs based on spatial and temporal baseline thresholds. By focusing on pairs with shorter intervals and closer spatial alignment, SBAS-InSAR effectively minimizes atmospheric noise and mitigates temporal and spatial decorrelation issues associated with conventional long-baseline PS-InSAR [18,19].

In recent years, driven by China’s rapid urbanization, mountain excavation and city construction (MECC)—commonly referred to as “cutting mountains and filling gullies”—has emerged as a critical urban development strategy in land-scarce regions of the loess hilly–gully terrain. Yan’an City, located in the core area of the Loess Plateau in Shaanxi Province, is surrounded by mountainous landscapes. Under the combined influence of national policies, economic growth, and rising urban demands, the shortage of land resources has become a major constraint on local economic and social development [20,21]. In response, the Yan’an New District (YND) initiated a MECC project as the cornerstone of its urban expansion plan, guided by the development principle of “relying on the old city, expanding along the river, and managing entire watersheds” [22]. This strategy represents a major step in addressing land scarcity while promoting coordinated regional development.

Although large-scale MECC projects can significantly increase the flat land area available for urban expansion, they inevitably have profound impacts on the local geological environment and may even induce new geological hazards [23]. The loess hilly–gully region surrounding Yan’an is characterized by complex and variable geological and hydrogeological conditions. Extensive land reclamation and rapid urban development have led to the compaction of loess fill foundations, causing significant and uneven subsidence [24]. Yan’an is located within the hilly and deeply incised gully landscape of the Loess Plateau. Large-scale operations such as mountain cutting, gully filling, and land reclamation have rendered the area susceptible to uneven land subsidence. This vulnerability stems from the inherent properties of loess—specifically, its large pores, metastable microstructure, and strong sensitivity to moisture. Under dry conditions, loess exhibits high permeability; however, water infiltration causes pore collapse and rapid strength reduction. The microstructure softens and reorganizes after wetting, further decreasing its load-bearing capacity [25]. Consequently, the combination of extensive MECC activities and prolonged rainfall has resulted in uneven land subsidence across the YND. Such subsidence not only jeopardizes building safety and longevity but also threatens urban infrastructure and residents’ daily lives. Therefore, identifying the factors driving severe subsidence in the district is a critical technical challenge that requires urgent attention.

The MECC project in YND has been ongoing for an extended period and spans a large area. Traditional geodetic techniques are inadequate for the frequent and rapid acquisition of deformation data in this region. Previous studies employing the SBAS-InSAR technique have documented widespread surface subsidence following the implementation of MECC projects [26], confirming the method’s applicability and reliability for monitoring land subsidence in YND due to the significant land subsidence caused by MECC projects in the study area during the research period. Large spatial baselines may lead to decorrelation. Therefore, the SBAS-InSAR technique was selected in this study to analyze the deformation in the region. Current research primarily focuses on the spatiotemporal evolution of subsidence [27], the effects of anthropogenic factors [28], mechanisms of uplift [29], and vegetation restoration characteristics [30]. Meanwhile, variations in groundwater storage have also been shown to influence loess subsidence in the area [31,32]. However, systematic and in-depth investigations into the role of natural factors—particularly the relationships among precipitation, groundwater storage, and the spatial distribution of subsidence after MECC completion—remain limited. Fluctuations in groundwater storage are recognized as a major driver of land subsidence. These changes not only affect the stability and compressibility of foundation soils but also alter soil structure and surface morphology over time. The effects are especially significant in areas of fill and excavation. Nevertheless, existing studies have not fully revealed the specific mechanisms or quantified the relationship between groundwater changes and land subsidence in YND. Therefore, a more comprehensive and systematic study is urgently needed.

Based on the above background, this study employs the SBAS-InSAR technique to further investigate the factors contributing to land subsidence in YND following the completion of MECC project. It systematically analyzes key influencing variables, including groundwater storage and fill thickness. Groundwater storage primarily governs the temporal evolution of deformation, while fill thickness determines its magnitude and spatial distribution. Sentinel-1A data were selected, and SBAS-InSAR processing was applied to obtain subsidence information spanning five years after the completion of major engineering projects. Using the derived deformation data, the study focuses on analyzing the spatial distribution patterns of subsidence and evaluating the respective impacts of groundwater storage and fill thickness. This in-depth investigation provides a scientific basis for improving subsidence control and prevention strategies in future land development efforts.

## 2. Materials and Methods

### 2.1. Study Area

**Figure 1:**
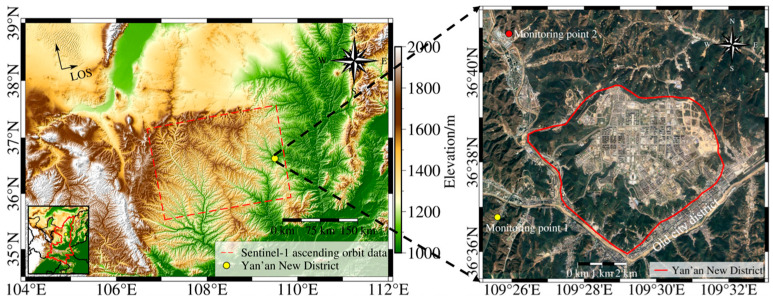
Location of the study area (**left**) and development status of YND in 2022 with the district boundary (**right**).

YND is situated in the northern part of Shaanxi Province, at the center of the Loess Plateau. The original topography surrounding the old city district features a hilly and gully landscape characterized by significant elevation changes. The watershed is approximately 1200 m above sea level, while the valley floor is around 1000 m, resulting in an elevation difference of about 200 m. The old city district of Yan’an is linearly distributed in a “Y” shape along the river valley, as shown in Figure 1. This Figure also indicates the geographical locations of two national groundwater monitoring points near YND.

YND encompasses a total planned area of 78.5 km^2^. The development of the new district primarily relies on the “land reclamation and city-building” project, which transforms the gully landscape of the Loess Plateau into flat areas suitable for urban construction [33]. Construction began in April 2012, and the geotechnical engineering phase was officially completed in May 2017, covering an area of 22.3 km^2^. The total earthwork volume for the project reached several hundred million cubic meters. As YND continues to develop, land subsidence issues have gradually emerged, displaying heterogeneous characteristics across different areas. These subsidence issues are closely related to factors such as fill thickness and soil compressibility.

This region experiences a temperate continental monsoon climate with distinct seasons, characterized by an average annual temperature of approximately 10 °C. Precipitation is primarily concentrated between May and August, with an average annual rainfall of about 500 to 600 mm. Groundwater extraction in YND is limited, with the area’s groundwater mainly recharged by atmospheric precipitation [34]. The land reclamation and city-building project has disrupted the original loess and hydrogeological conditions, leading to subsidence phenomena in the area following construction [35].

### 2.2. Data Sets

This study primarily investigates the long-term subsidence in YND after its completion. Due to large-scale excavation during the early stages of the project (2014–2016), land subsidence was pronounced. The extensive excavation led to phase decorrelation. To mitigate this issue, Sentinel-1 SAR data from 2017 onward were selected for the study. Currently, Sentinel-1 consists of the Sentinel-1A satellite and Sentinel-1C satellite, which carry a C-band synthetic aperture radar (SAR). The Sentinel-1A satellite was launched on 3 April 2014, and the Sentinel-1C satellite was launched on 5 December 2024, with a revisit cycle of 12 days. Its imaging mode is the interferometric wide swath (IW) mode, with a spatial resolution of 5 × 20 m and an incidence angle of 29~46°.

**Figure 2:**
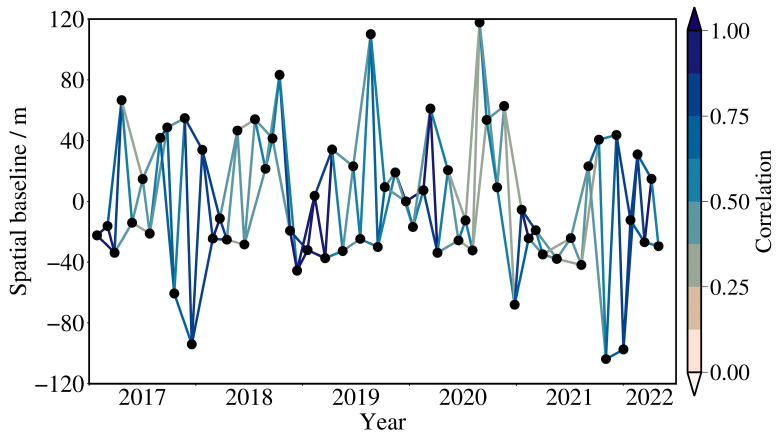
Spatio-temporal baseline of the interferometric pairs.

A total of 66 ascending Sentinel-1A radar images were used in this study, covering the period from 28 January 2017 to 2 May 2022. The polarization mode used was VV polarization. Figure 2 shows the spatiotemporal baseline of the selected images. To reduce the impact of orbital errors, precise orbital data for Sentinel-1A were selected. This study selected the Copernicus Digital Elevation Model (COP DEM), which was developed under the European Union’s Copernicus Programme and produced by the European Space Agency (ESA). The dataset has a spatial resolution of 30 m, and it was used in this study to correct terrain errors. These data also served as the original topographic data for the region before the MECC project, allowing the analysis of deformation rate trends in areas of fill and excavation. Additionally, the global atmospheric correction observation system’s atmospheric delay phase model was used for atmospheric correction to reduce the influence of atmospheric delay on the data [36].

To study the impact of natural factors on subsidence in YND, the precipitation data of YND from 2017 to 2022 were obtained from the 1 km monthly precipitation dataset provided by the National Tibetan Plateau Data Center of China [37]. This data is used to analyze the relationship between precipitation, time-series subsidence, and groundwater storage. Groundwater storage data is sourced from the GLDAS Catchment Land Surface Model V2.2 (GLDAS CLSM V2.2) dataset. This dataset integrates land hydrological processes simulated by the CLSM model with gravity field data from the GRACE satellite, generated through data assimilation, and provides global spatiotemporal information on water cycle variables such as soil moisture, evapotranspiration, and groundwater storage, with a resolution of 0.25° × 0.25° per day [38]. For this study, groundwater storage data corresponding to the InSAR image dates was extracted from this dataset. Considering the relatively low spatial resolution of the GLDAS CLSM V2.2 dataset, groundwater storage data for YND were validated using early groundwater monitoring points in YND from previous studies [39] and groundwater monitoring data provided by the China Geological Environmental Monitoring Institute to ensure the suitability of this dataset for the study area.

To analyze terrain changes in the study area over different periods, this study selected two DEM datasets acquired at different times: the ZY-3 satellite-derived DEM and the Advanced Spaceborne Thermal Emission and Reflection Radiometer Global Digital Elevation Model (ASTER GDEM). The ASTER GDEM dataset was acquired before the construction period of YND but after the Shuttle Radar Topography Mission (SRTM) DEM; therefore, ASTER GDEM was differenced with the ZY-3 DEM to analyze elevation changes in the study area. The ZY-3 DEM was generated from 2019 ZY-3 satellite imagery through stereoscopic mapping to produce a high-resolution digital elevation model. The ZY-3 DEM has a spatial resolution of 10 m and a vertical accuracy better than 5 m, while the ASTER GDEM has a spatial resolution of 30 m and a vertical accuracy of approximately 10–20 m.

**Figure 3:**
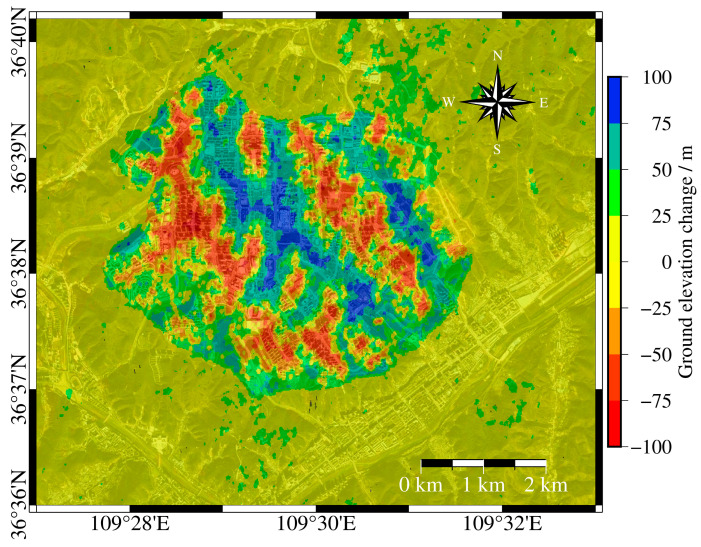
Elevation difference obtained by subtracting ASTER GDEM from ZY-3 DEM, representing the thickness of fill and excavation in YND. Positive values indicate fill thickness, while negative values indicate excavation thickness.

Given the difference in spatial resolution between the two DEMs, preprocessing was necessary before elevation differencing. Specifically, the ASTER GDEM was resampled to match the 10-m resolution of the ZY-3 DEM using bilinear interpolation to maintain continuity of elevation values. After co-registration and resampling, elevation differences between the two DEMs were calculated on a pixel-by-pixel basis to obtain terrain changes in the region (see Figure 3).

### 2.3. Methodology

After the completion of the MECC project in YND, there was a lack of permanent scatterer points in the short term. Therefore, in this study, SBAS-InSAR technique was used to monitor surface deformation in YND [40]. InSAR Scientific Computing Environment (ISCE, version 2.6.4) software was applied for interferometric processing. The specific parameters for selecting the interferometric pairs are as follows: maximum temporal baseline of 36 days, spatial baseline of 150 m, minimum coherence of 0.3, and a line-of-sight to azimuth ratio of 4:1. Goldstein filtering was applied to suppress interference noise [41]. To reduce orbital and atmospheric delay errors, precise orbital data and GACOS data were used for correction [42]. Phase unwrapping was performed using the Minimum Cost Flow (MCF) network method [43]. The data were processed using the Miami InSAR Time-series software in Python (MintPy, version 1.6.2) software for time-series analysis [44], and the time-series deformation in the line-of-sight (LOS) direction was extracted through the Singular Value Decomposition (SVD) algorithm. The deformation values presented in this study are referenced to the image from 28 January 2017. The stable reference points were selected based on:

(1) High-resolution satellite imagery was used to identify areas without significant surface changes or construction activities during the study period;
(2) Preliminary InSAR results were then used to select pixels within these areas that exhibited minimal deformation (cumulative displacement < 2 mm, standard deviation < 1 mm) and stable time series behavior.

The large-scale subsidence in YND is primarily due to the MECC project, which mainly results in displacements in the vertical direction. However, the results obtained from InSAR monitoring represent displacements along the radar line of sight (LOS). Therefore, it is necessary to project the displacements along the radar line of sight into the vertical direction using the radar incidence angle. The conversion can be performed using the following formula:

(1) $$d_{v}=\frac{d}{cos\theta }$$

where, $d_{v}\;$ is the displacement in the vertical direction at the observation point, d is the displacement along the radar line of sight, and θ is the radar incidence angle, the radar incidence angle in the study area is 39°. In this study, the cumulative deformation and deformation rate along the radar line of sight are projected into the vertical direction for analysis using Formula (1).

In Section 3.2, a fifth-order polynomial function was employed to fit the deformation rate curves. The temporal variation in deformation rate exhibits strong nonlinearity, influenced by multiple factors such as precipitation, mining activities, and geological structures. As a result, the rate curves display complex trends, including multi-peak and fluctuating patterns. To capture these characteristics effectively, a fifth-order polynomial was selected for curve fitting. The general form of the fifth-order polynomial function is expressed as:

(2) $$f\left(x\right)=a_{5}x^{5}+a_{4}x^{4}+a_{3}x^{3}+a_{2}x^{2}+a_{1}x+a_{0}$$

where $f\left(x\right)$ denotes the fitted function, x represents the surface deformation rate, and $a_{0},\;a_{1},\;a_{2},\;a_{3},\;a_{4},\;a_{5}\;$ are the fitting coefficients determined using the least squares method.

In Section 4.2, the correlation curve between groundwater content and cumulative deformation was fitted using the Kernel smoothing method. Kernel smoothing is a non-parametric regression technique that estimates a smooth curve by locally weighting nearby data points using a kernel function (e.g., Gaussian kernel). Since the relationship between groundwater content and cumulative deformation does not necessarily follow a known parametric form and may exhibit nonlinear but implicit patterns, kernel smoothing provides a flexible and effective means for curve fitting.

The kernel smoothing function is defined as:

(3) $$f\left(x\right)=\frac{\sum_{i=1}^{n}\;K_{h}\left(x-x_{i}\right)y_{i}}{\sum_{i=1}^{n}\;K_{h}\left(x-x_{i}\right)}$$

where $f\left(x\right)$ is the smoothed fitting curve, $K_{h}\left(\cdot \right)$ is the kernel function, h is the bandwidth parameter controlling the degree of smoothing, $x_{i}$ and $y_{i}$ are the positions and corresponding values of the observed data points, and n is the total number of data points. In this study, the Gaussian kernel was adopted, which is given by:

(4) $$K_{h}\left(x\right)=\frac{1}{\sqrt{2\pi h^{2}}}\exp\left(-\frac{x^{2}}{2h^{2}}\right)$$

By adjusting the bandwidth parameter h, the smoothness of the fitted curve can be controlled. A smaller h yields a curve that closely follows the original data points, while a larger h results in a smoother but more generalized fit.

Section 4 selects several subsidence points for detailed analysis and selects the main study areas based on the thickness of fill and excavation. The areas with fill thickness greater than 50 m are primarily selected from the Gaojiagou and Qiaoergou regions. Additionally, considering that filling activities occurred in the Tanyaogou area during the experimental period, the major fill zones of this area are also included in the analysis. Finally, through the monitoring results from these three regions, the study investigates the impact of groundwater storage and fill and excavation thickness on subsidence.

Monthly precipitation data over China at 1 km resolution provided by the National Tibetan Plateau Data Center of China is derived from the global 0.5° climate data published by the Climatic Research Unit (CRU) and the global high-resolution climate data published by WorldClim. The data was downscaled for China using the Delta spatial downscaling scheme. It was also validated using data from 496 independent meteorological observation points, and the validation results are reliable. To facilitate the comparison of time-series subsidence and precipitation changes, the precipitation data for YND was averaged to obtain the monthly precipitation data for YND.

In Section 4.1, the analysis of the impact of precipitation requires considering the cumulative effect of precipitation over different periods when calculating the deformation rate. Since the impact of precipitation on cumulative deformation is not an immediate response but gradually becomes apparent after continuous precipitation, the cumulative deformation from multiple observation points and time-series precipitation data can be combined using the following method during data processing: First, determine a period of three consecutive months that corresponds to a specific precipitation range, and then extract the cumulative deformation for the subsequent three months. This approach is used to calculate the deformation rate for different precipitation periods.

The groundwater storage data obtained from the GLDAS CLSM V2.2 dataset is point data. To conduct a spatial correlation analysis with the InSAR monitoring point data, the kriging interpolation method is used to spatially interpolate the groundwater storage data, converting it into a spatiotemporal sequence corresponding to the InSAR monitoring points in order to obtain the groundwater storage for each InSAR point.

When analyzing the correlation between groundwater content and time-series surface deformation, it is important to consider that groundwater variations may influence land subsidence with a certain time lag [45]. Therefore, in Section 4.2 of this study, cumulative deformation data were matched with groundwater content data from previous months to account for potential lag effects. The correlation was then quantified using the Pearson correlation coefficient, which is calculated as follows:

(5) $$r=\frac{\sum_{i=1}^{n}\;\left(x_{i}-\bar{x}\right)\left(y_{i}-\bar{y}\right)}{\sqrt{\sum_{i=1}^{n}\;{\left(x_{i}-\bar{x}\right)}^{2}\sum_{i=1}^{n}\;{\left(y_{i}-\bar{y}\right)}^{2}}}$$

where $x_{i}$ and $y_{i}$ are the i-th elements of vectors x and y, respectively; $\bar{x}$ and $\bar{y}$ represent the mean values of the corresponding vectors; and n is the total number of elements in the vectors.

When calculating the correlation between groundwater content with a time lag and cumulative surface deformation, vectors x and y represent the cumulative deformation data and groundwater content data with a given lag, respectively, while n denotes the number of points within the study area. Correlation coefficients were computed for lag periods ranging from 1 to 6 months. The results of this multi-lag correlation analysis indicate that land subsidence exhibits the strongest correlation with groundwater content lagged by two months. Therefore, a two-month lag was adopted in this study, meaning that the subsidence value for a given month was matched with the groundwater content from two months earlier. For instance, the land subsidence in March 2017 was matched with the groundwater content from January 2017 for correlation calculation.

In the exploration of the relationship between the thickness of fill and excavation and the deformation rate, representative characteristic points within the study area are selected to analyze their time-series deformation features. The focus is on the duration of the primary and secondary consolidation stages after the filling and excavation project and their impact on land subsidence. These stages are considered the primary human influence factors on subsidence in YND, providing a deeper understanding of the control mechanisms of filling and excavation projects on surface deformation.

## 3. Results

### 3.1. Ground Deformation Information Spatial Distribution

**Figure 4:**
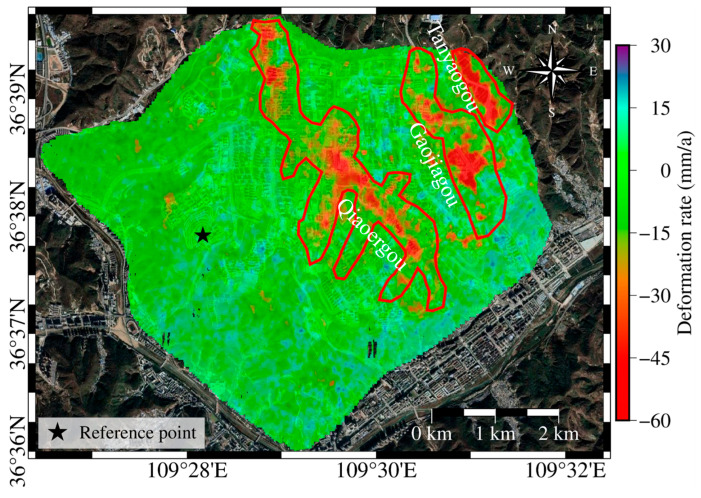
Surface deformation in YND from 28 January 2017 to 2 May 2022, showing the surface deformation rates across the study area during the observation period. Three significant subsidence zones are outlined with red boundaries, and stable reference points were selected based on satellite imagery. Positive values indicate surface uplift, while negative values indicate land subsidence.

The SBAS-InSAR technique was used to process Sentinel-1A data from the study area, spanning from 28 January 2017 to 2 May 2022, to obtain a long-term time series of land subsidence information for YND (see Figure 4). Since the land subsidence caused by the high fill areas of loess is mainly in the vertical direction, the displacement along the radar line of sight is projected to the vertical direction using Formula (1) for better analysis of the subsidence in the area. The positive and negative values in the figure represent the vertical displacement rate, with positive values indicating uplift and negative values indicating subsidence. Stable deformation points are selected as reference points for the deformation analysis.

The main subsidence areas in YND are concentrated in the Qiaoergou and Gaojiagou regions. In the northwest of Qiaoergou, subsidence rates range from −16 to −50 mm/a, with a maximum rate of −63 mm/a. In the original Qiaoergou area, now covering Luyi Ecological Park to Yan’an People’s Park, the subsidence rate ranges from −10 to −50 mm/a, with a maximum of −76 mm/a. The Gaojiagou area also exhibits extensive subsidence, with rates between −10 and −30 mm/a and a peak value of −65 mm/a. In the Tanyaogou area, based on Google Earth imagery, earthwork operations began in April 2018 and were mostly completed by August 2018. During the study period, this region exhibited subsidence rates ranging from −20 to −70 mm/a, with a maximum rate of −89 mm/a.

### 3.2. Subsidence Characteristics and Excavation-Filling Engineering Correlation Analysis

**Figure 5:**
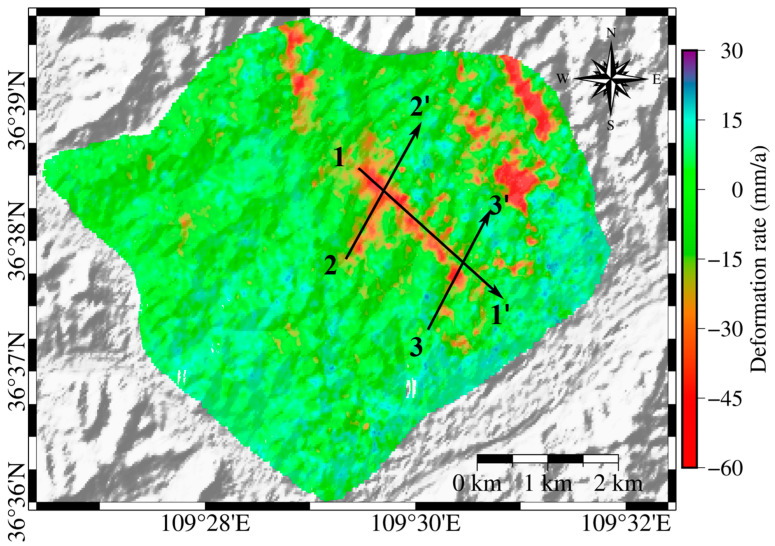
Surface deformation rates overlaid on the ASTER GDEM to analyze the relationship between deformation rates and the original terrain. Three prominent deformation profiles are marked to facilitate subsequent analysis of the correlation between deformation rates and the original topography along the profiles.

**Figure 6:**
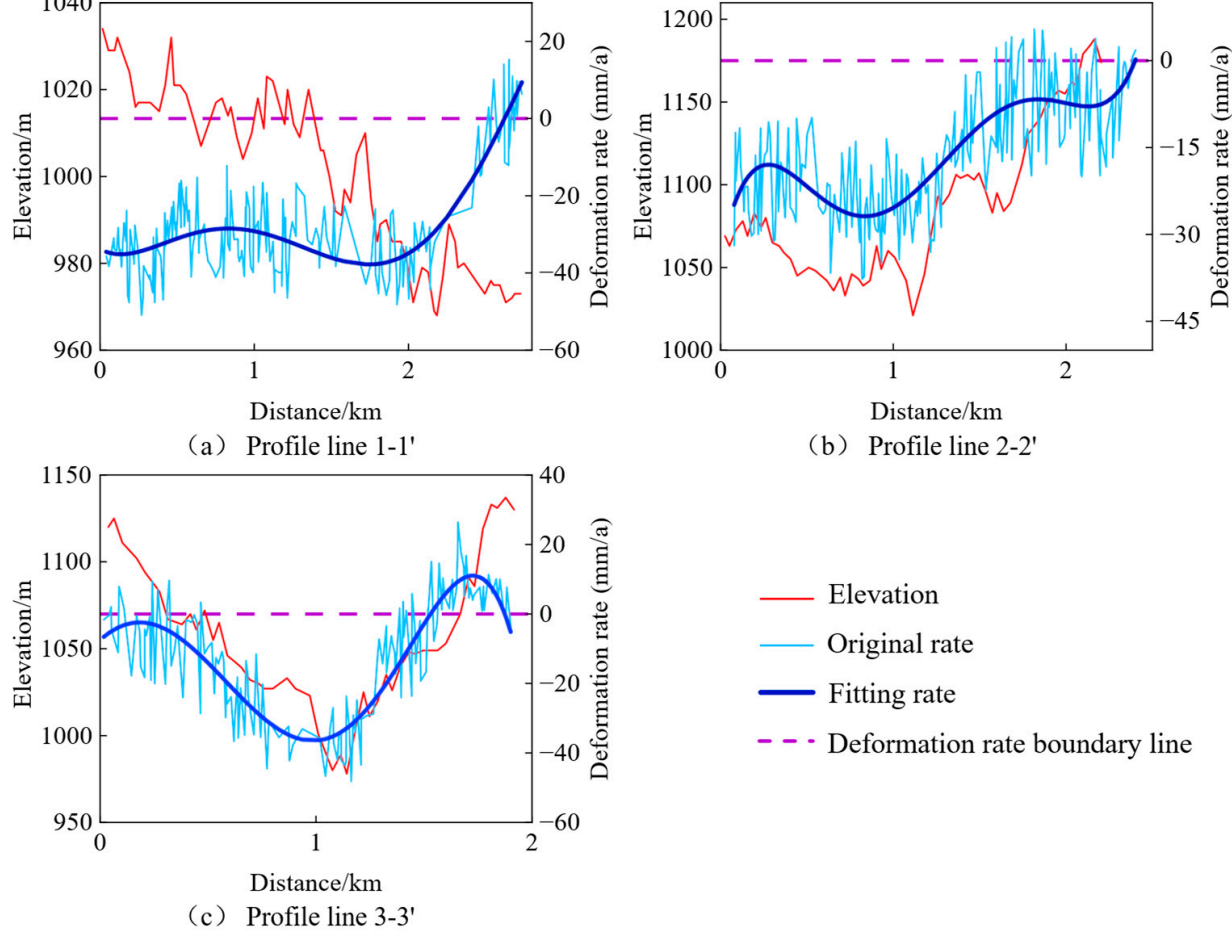
Relationship between Elevation and Deformation Rate along Three Profiles. (**a**) Profile line 1–1′; (**b**) Profile line 2–2′; (**c**) Profile line 3–3′.

Figure 5 presents the deformation rate map overlaid with the 30 m resolution SRTM DEM and includes profile lines across areas with pronounced subsidence, with arrows indicating the direction of profile extraction. The three sets of numerical labels correspond to the three deformation rate profiles shown in Figure 6. As evident in Figure 5, the most pronounced subsidence areas are primarily located in valley regions, which served as fill areas during construction. YND is situated on the Loess Plateau, where the pre-construction geological composition is dominated by Quaternary loess from the Cenozoic era. During the MECC process, loess from cut areas was used to fill the valleys. This fill loess was subjected to compaction, disrupting the original soil structure and weakening the cementation and bonding strength between loess particles. As a result, noticeable subsidence has occurred in these fill zones [46].

Three areas with significant topographical changes were selected from Figure 5 for deformation rate profile analysis, as shown in Figure 6. In Figure 6, the purple dashed line indicates the boundary where the deformation rate is zero. The detailed analysis is as follows:

1. Analysis of profile 1–1′: This area is situated in the valley region, with the terrain gradually descending to the south. As shown in Figure 6a, significant subsidence is evident in the fill area, while only the unconstructed section at the end demonstrates a stable condition.
2. Analysis of profile 2–2′: The first half, representing the fill area, shows significant subsidence, while the latter half, the excavation area, exhibits a tendency toward stability.
3. Analysis of profile 3–3′: This area exhibits topographical changes in a mountain-valley-mountain pattern. The excavation areas on the peaks demonstrate relatively stable conditions, while the fill area in the valley shows significant subsidence.

Such deformation may be related to structural weakening of the loess caused by fill loading and variations in water content [35]. This results in varying degrees of subsidence due to external factors, while the excavation areas exhibit a slight upward trend.

**Figure 7:**
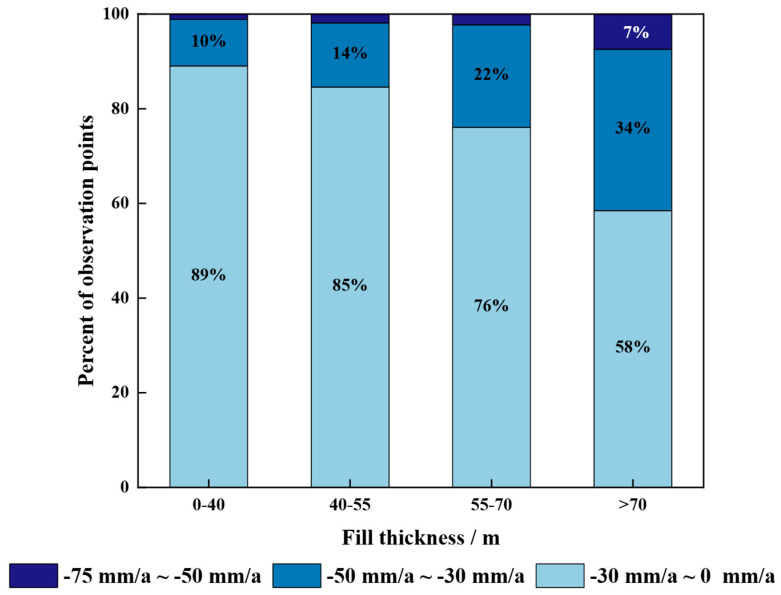
Relationship between subsidence rate and fill thickness. The proportions shown in the figure represent the distribution of different subsidence rates under varying fill thickness conditions.

An analysis combining excavation/fill thickness and deformation rates in the Qiaoergou area (see Figure 7) reveals that zones with greater fill thickness tend to have a significantly higher number of observation points exhibiting moderate to high subsidence rates (−75 to −30 mm/a). In contrast, areas with relatively thin fill layers are primarily associated with subsidence rates ranging from −30 to 0 mm/a. This suggests that subsidence severity increases with fill thickness, which is consistent with the fundamental principle that greater fill thickness leads to larger compression magnitudes.

## 4. Analysis

### 4.1. Influence of Precipitation

**Figure 8:**
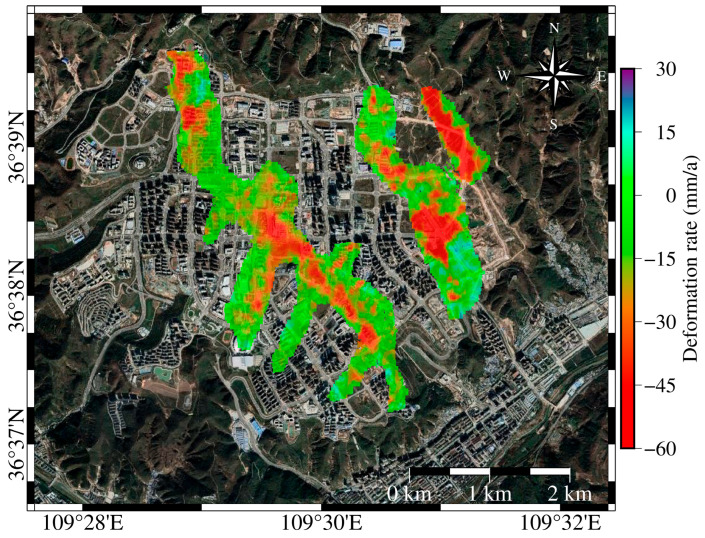
Subsidence rates extracted from the three significant deformation zones identified in Figure 4.

The areas exhibiting notable subsidence are primarily composed of filled loess, which is known for its distinct water sensitivity. In the Loess Plateau region, where the climate is arid, surface water resources mainly rely on precipitation recharge. Therefore, precipitation plays a crucial role in influencing subsidence in these areas. Based on the method for extracting major deformation points described in Section 2.3, the relevant data points were filtered, and the deformation rate points were overlaid on Google Earth imagery (see Figure 8) to visually illustrate the land subsidence across these regions.

**Figure 9:**
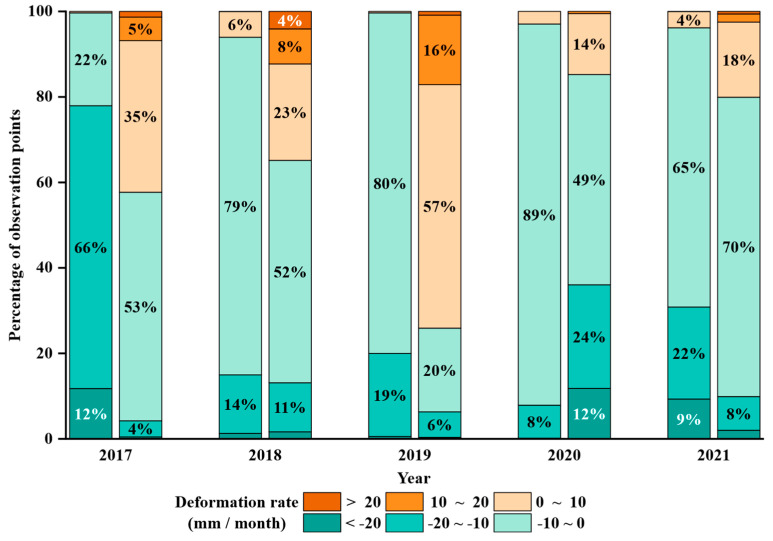
Relationship between Precipitation and Deformation Rate. Each year includes two columns: the left column represents the proportion of different deformation rates under high precipitation, while the right column represents the proportion under low precipitation.

To investigate the impact of precipitation on surface deformation, surface deformation rates during periods of high precipitation (≥60 mm/month) and low precipitation (<60 mm/month) were calculated based on cumulative displacement, as outlined in Section 2.3. The statistical analysis results are presented in Figure 9, which shows that the surface deformation rates differ significantly between the two precipitation conditions. For each year, the left-hand data represent the high-precipitation period, while the right-hand data correspond to the low-precipitation period. Overall, high precipitation periods are more likely to induce larger surface displacements, while deformation rates during low precipitation periods are relatively lower. In most cases, high precipitation periods correspond to increased subsidence compared to low precipitation periods, suggesting that precipitation may accelerate the deformation process. During low precipitation periods, a larger proportion of deformation rates fall within the range of −10 to 10 mm/month, implying that reduced rainfall may slow down surface displacement or even lead to stabilization.

### 4.2. Impact of Groundwater Storage

The excavation and filling project caused changes in the structure of the loess. Loess exhibits sensitivity to water resources, and the infiltration of groundwater into the altered loess layer can lead to loess subsidence, compression, and deformation, thereby accelerating land subsidence. According to the analysis in Section 4.1, the impact of precipitation on areas with significant subsidence shows a certain lag, and precipitation also has a certain effect on groundwater storage [47].

**Figure 10:**
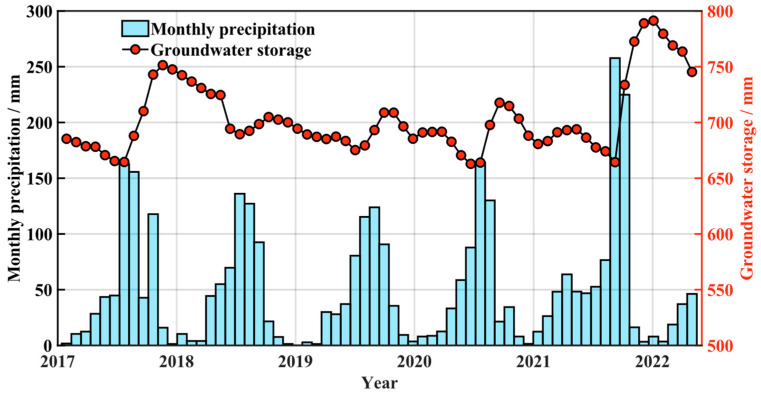
Relationship between Precipitation and Groundwater Storage. The left vertical axis represents monthly precipitation, and the right vertical axis represents groundwater storage. The blue bar chart illustrates the variation in precipitation, while the red solid line with markers shows the change in groundwater storage.

Precipitation in the region is mainly concentrated in the summer months from May to August, with a relatively short rainy season. As shown in Figure 10, it is clear that groundwater storage gradually increases 1–3 months after a significant rise in precipitation, peaking within 2–3 months. When precipitation starts to increase, groundwater storage does not immediately reflect the change in precipitation; however, when precipitation begins to decrease, groundwater storage gradually increases. During the drier seasons, groundwater storage shows a continuous decreasing trend. In conclusion, there is a certain relationship between precipitation and groundwater storage, with groundwater showing a delayed response to precipitation. This lag not only affects the dynamic changes in groundwater but also influences land subsidence, making it one of the important natural factors impacting ground settlement.

**Figure 11:**
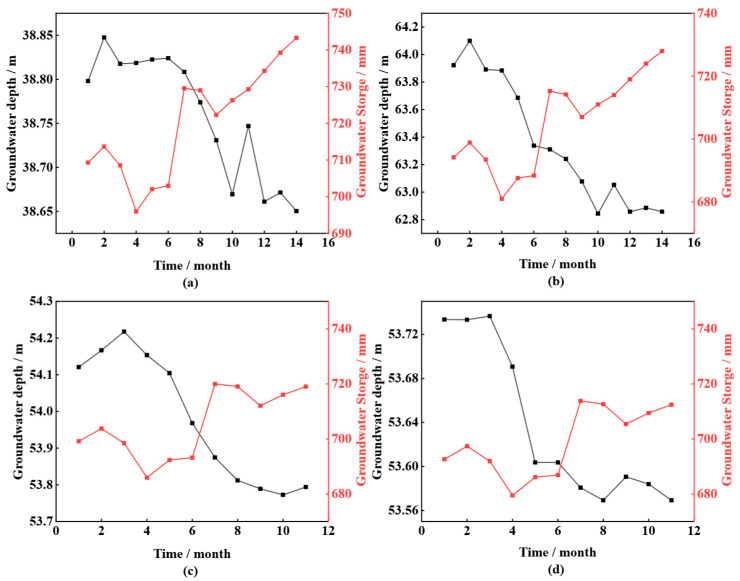
Relationship between Groundwater Level and Groundwater Storage in Previous Studies. (**a**) Early Monitoring Site 1; (**b**) Early Monitoring Site 2; (**c**) Early Monitoring Site 3; (**d**) Early Monitoring Site 4.

To verify the trend of groundwater storage and land subsidence, this study first selects four groundwater monitoring points in the Qiaoergou area, which were set up during the engineering period as part of previous research, to validate the groundwater storage data obtained from the GLDAS CLSM V2.2 dataset. These monitoring points were established between 2014 and 2015, during a period of frequent excavation and filling activities, resulting in significant groundwater level fluctuations. This serves to monitor the groundwater level in the Qiaoergou area. Figure 11 compares the changes in groundwater levels and groundwater storage at different monitoring points.

**Figure 12:**
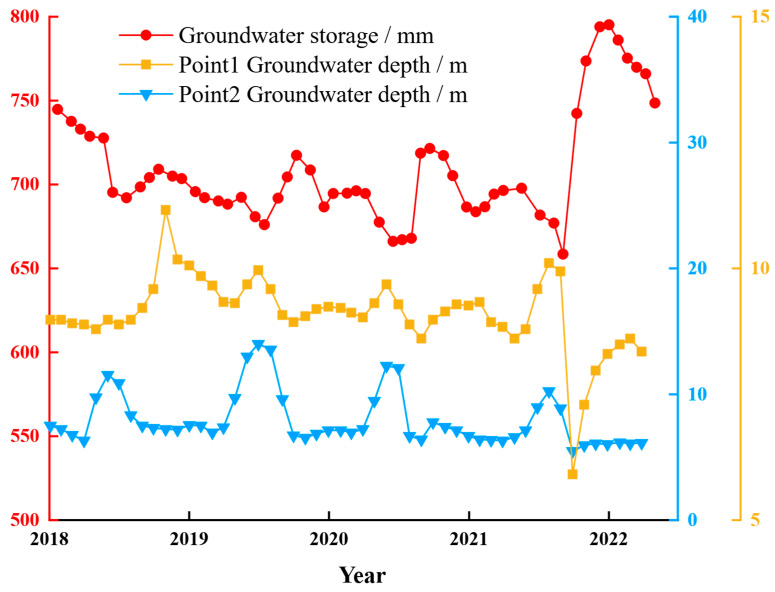
Comparison between Groundwater Level Data from National Groundwater Monitoring Stations and the GLDAS CLSM V2.2 Dataset in Recent Years. The left red vertical axis represents groundwater storage from the GLDAS CLSM V2.2 dataset. The right blue vertical axis corresponds to the groundwater level at Monitoring Site 2, and the right yellow vertical axis corresponds to the groundwater level at Monitoring Site 1. In the figure, the red solid line with dots shows the groundwater storage data, the blue solid line with triangles indicates the groundwater level at Monitoring Site 2, and the yellow solid line with squares represents the groundwater level at Monitoring Site 1.

Due to the lack of recent monitoring data from earlier monitoring points, the groundwater storage data from the GLDAS CLSM V2.2 dataset was validated using recent groundwater monitoring data provided by the China Geological Environment Monitoring Institute. Figure 12 presents the comparison and analysis results of groundwater monitoring point data and groundwater storage data from 2018 to June 2022.

The groundwater level is equal to the wellhead elevation minus the depth to groundwater, and since the wellhead elevation is constant, there is an inherent correlation between groundwater level and depth to groundwater. Therefore, both the monitoring points from previous studies and the two national groundwater monitoring points show a significant negative correlation between groundwater storage and depth to groundwater, meaning that as groundwater storage increases, the groundwater level decreases. This trend is generally consistent across different time periods and monitoring points. Previous studies have further confirmed the significant negative correlation between groundwater storage and depth to groundwater [48]. In conclusion, the trend of groundwater storage changes from the GLDAS CLSM V2.2 dataset is highly consistent with the groundwater monitoring data from the China Geological Environment Monitoring Institute, indicating that this dataset can be used to study the correlation between groundwater storage and surface deformation in YND.

Based on Figure 11 and Figure 12, it can be observed that the changes in groundwater level are only a few meters, but their impact on the compression of the fill layer should not be underestimated. Firstly, since the fill material is primarily loess, these changes will still have a significant effect on the compression of the fill; these variations are periodic or repetitive, and they could lead to gradual compression or expansion of the soil, thus affecting the stability and settlement of the fill. The compressibility of loess and its high sensitivity to moisture changes mean that even fluctuations in groundwater levels of just a few meters could trigger the expansion or contraction of loess, leading to settlement or compression of the fill.

Based on the deformation rate map presented in Figure 10, the deformation rate ranges are defined as follows: Areas with deformation rates between 5 and −5 mm/a are considered stable. Areas with subsidence rates between −5 and −20 mm/a are categorized as regions with relatively low subsidence rates. Areas with deformation rates greater than −20 mm/a are classified as regions with high subsidence rates.

**Figure 13:**
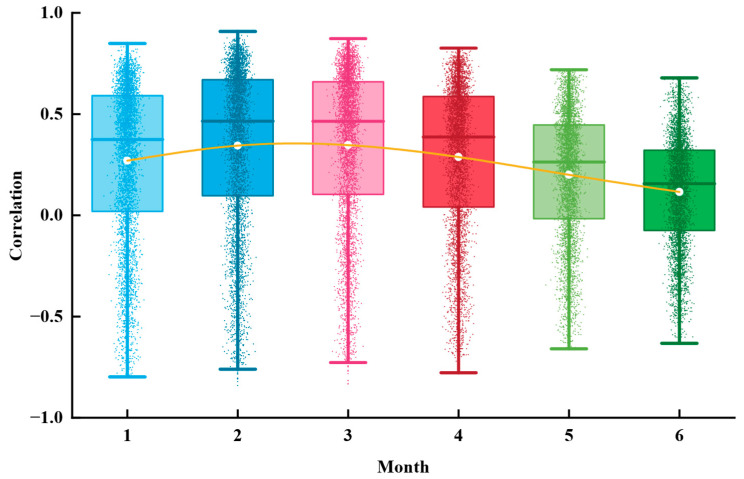
Correlation Analysis between Groundwater Storage and Cumulative Deformation with 1–6-Month Lag. Groundwater storage is matched with cumulative deformation data delayed by 1 to 6 months, and the correlation between them is calculated for each lag period. The horizontal axis represents the number of lag months, and the vertical axis shows the Pearson correlation coefficient.

To explore the relationship between groundwater storage and cumulative deformation, and considering the lag effect of deformation data relative to groundwater data, this study calculated the correlation between deformation data and groundwater storage data with a lag of 1 to 6 months (see Figure 13). In the figure, the white points represent the correlation averages for different lag months, and the yellow lines connect these averages to visually present the trend of correlation changes with lag time. The results show that the highest correlation occurs with a lag of 2 months, with both the average and median values outperforming those for other lag months. Therefore, for the analysis of the relationship between groundwater storage and cumulative deformation in YND, a lag of 2 months is used for correlation analysis.

**Figure 14:**
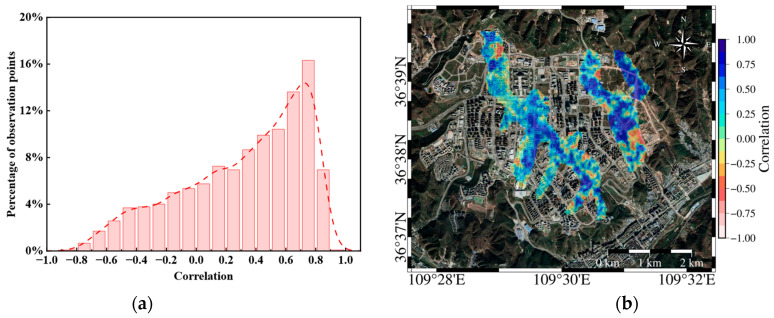
(**a**) Correlation chart of groundwater storage and deformation; (**b**) Mapping of groundwater storage and deformation correlation on Google Earth Image.

We matched the cumulative deformation data with the groundwater storage, with a two-months lag, and calculated the correlation, and calculate the correlation between the cumulative deformation at each point in the significantly subsiding areas and the groundwater storage (see Figure 14). Figure 14a uses a kernel smooth distribution fitting curve to fit the correlation frequency. From the results in Figure 14a, the overall correlation range in the significantly subsiding areas is between −0.8 and 0.8, with a large amount of data showing correlations between 0.4 and 0.8. Many subsiding points exhibit a positive correlation, indicating that the deformation data is consistent with the groundwater storage data from two months ago. In Figure 14b, the correlation between the deformation at each point and the groundwater storage is shown on the Google Earth image.

**Figure 15:**
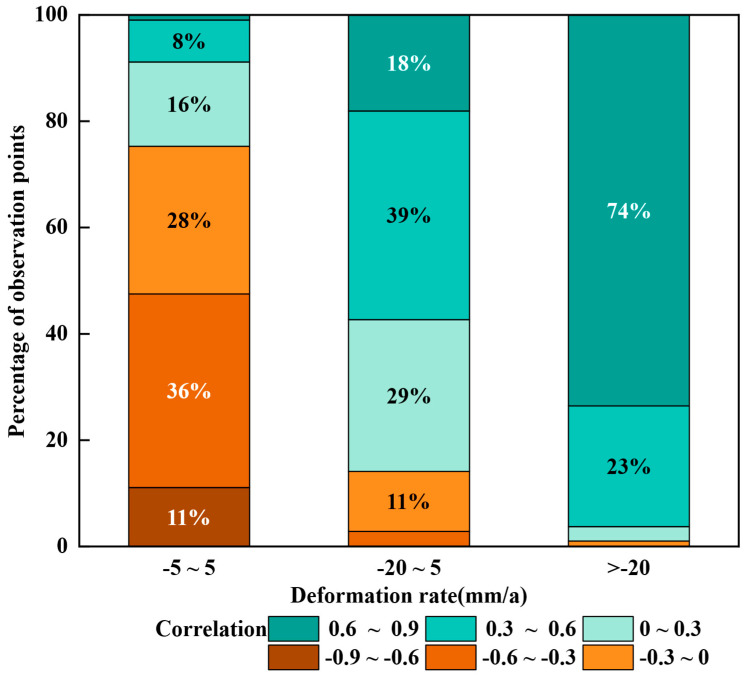
Proportion of Correlation Levels within Different Deformation Rate Intervals. Shows the proportion of different correlation levels within each deformation rate interval. The horizontal axis represents various deformation rate intervals, and the vertical axis indicates the proportion of each correlation level.

Combining Figure 8 and Figure 14b, there is a certain correlation in the spatial distribution between groundwater storage and surface deformation. A statistical analysis was conducted on the correlation between lagged cumulative deformation values and groundwater storage at all deformation rate points in the main study area (see Figure 15).

In areas with lower deformation rates, such as the southern part of Gaojiagou and the western part of Qiaoergou, the correlation between the corresponding cumulative deformation and groundwater storage shows a strong negative correlation. Combining the elevation change map before and after the project in Figure 3 and the SRTM elevation data in the base map of Figure 5, it is speculated that the elevation of these areas is relatively high, with excavation as the primary activity. This did not cause significant damage to the original loess structure. Surface water infiltrates through the gaps in the loess, forming groundwater. As the original loess structure is stable and has strong permeability, groundwater storage is better able to reflect the relationship with surface deformation. In regions with stable deformation rates, it is speculated that there is no consolidation stage triggered by the amount of engineering work, making surface deformation more sensitive to changes in groundwater storage. The correlation between cumulative deformation and groundwater storage for the current month shows a positive correlation. However, when calculating the correlation, the groundwater storage data is matched with the cumulative deformation data lagged by two months, resulting in a negative correlation in the calculated results. In regions with high subsidence rates, the positive correlation between groundwater storage and cumulative deformation is more pronounced. For example, in areas with significant subsidence in Qiaoergou and Gaojiagou, the correlation between cumulative deformation and groundwater storage is relatively high. It is speculated that these areas were originally valleys with large fill thickness, and they were affected by the loess consolidation stage for a long period of time. However, the construction period has been completed for quite a while, and most of these areas have gradually entered the natural subsidence stage. As a result, surface deformation is more influenced by natural factors such as groundwater, leading to a stronger positive correlation.

In Tanyaogou, the positive correlation is even more evident. This is because the area has a small amount of fill material and a short construction period, meaning the primary consolidation phase lasted only a short time. Before and after the primary consolidation phase, the influence of groundwater storage on surface deformation is more pronounced, which leads to a stronger positive correlation. It is speculated that in areas with large amounts of fill material, the gaps between loess particles are larger, reducing the permeability. As groundwater storage increases, the loess softens and causes land subsidence. Additionally, the reduced permeability leads to a delayed effect of groundwater storage on surface deformation.

In summary, in different areas of YND, the correlation between groundwater storage and surface cumulative deformation is significantly influenced by the amount of excavation and filling work. In areas with less excavation and filling or where no excavation and filling occurred, the correlation between groundwater storage and surface deformation is characterized by lower deformation rates and a strong negative correlation. This phenomenon is primarily due to the stability of the original loess structure and its higher permeability, which allows groundwater storage to more clearly reflect the relationship with surface deformation. On the other hand, in areas with larger fill thickness, due to changes in the loess structure and decreased groundwater permeability during the filling process, the settlement rate is higher, showing a positive correlation. This is likely because the loess in filled areas softens easily upon absorbing water, leading to significant surface settlement. Additionally, these regions enter a natural phase, where the influence of natural factors, such as groundwater storage, further strengthens, exacerbating the settlement. Therefore, the positive and negative correlations between the observed groundwater storage and surface deformation are closely related to the amount of excavation and filling work.

## 5. Discussion

### 5.1. Relationship Between Fill Thickness and Settlement Rate

**Figure 16:**
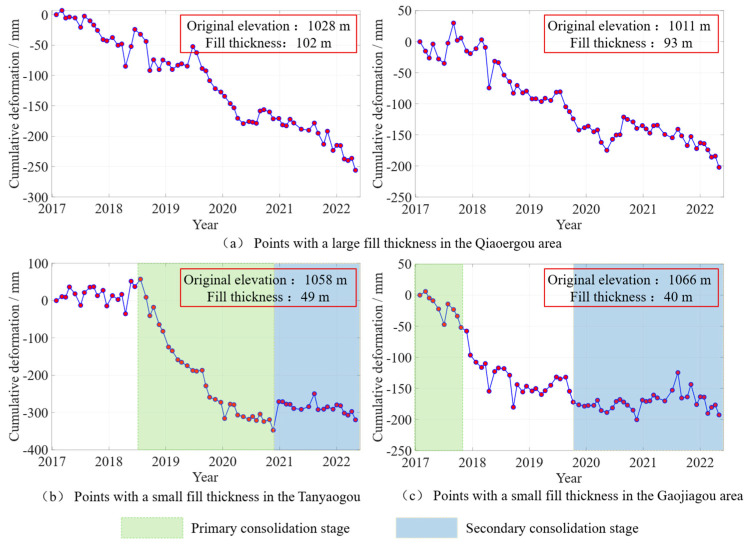
Condition of Filled Loess at Points with Different Original Elevations after Filling. (**a**) Points with a large fill thickness in the Qiaoergou area; (**b**) Points with a smal1 fil1 thickness in the Tanyaogou; (**c**) Points with a smal1l fil1 thickness in the Gaojiagou area.

Due to the inherent structural instability of the loess used in the fill bodies, varying fill heights significantly impact the soil’s consolidation state in the area [49]. By selecting representative points in areas with different elevations, Figure 16 presents an analysis of the relationship between fill body thickness and settlement rate.

Figure 16a shows two characteristic points located in the Qiaoergou area, which are in regions of significant subsidence with low original elevation. Although the construction period ended 3 years ago, the large fill thickness caused a prolonged and large subsidence rate during the natural settlement phase. Figure 16b features characteristic points located in the Tanyaogou area, where excavation and filling activities took place from April 2018 to September 2018. These points are in areas with higher original elevation and shorter construction periods, where the fill thickness is relatively small. Between April 2018 and April 2020, the points entered the primary consolidation phase with a significant increase in the subsidence rate, after which, post-April 2020, the points transitioned into a secondary consolidation phase with a reduced subsidence rate. Figure 16c shows characteristic points in the Gaojiagou area, which are also located in regions with higher original elevation and smaller fill thickness. Some engineering work had been done prior to the study period, and the area was still in the rapid subsidence phase of the primary consolidation. From November 2017 to January 2019, after the completion of road construction, the ground conditions gradually stabilized. After a period of rapid subsidence, the area entered the secondary consolidation phase with a reduced subsidence rate.

Comparative analysis of the data from the four points in the aforementioned areas reveals that fill body thickness significantly impacts the duration of the primary and secondary consolidation stages of filled loess. A thicker fill body prolongs the primary consolidation stage, extending the period of rapid surface settlement. In contrast, a thinner fill body shortens the primary consolidation phase, enabling an earlier transition to the secondary consolidation stage and faster stabilization of surface deformation. Consequently, fill bodies of varying thicknesses exhibit distinct effects on surface deformation.

### 5.2. YND Post-Completion Urban Planning

**Figure 17:**
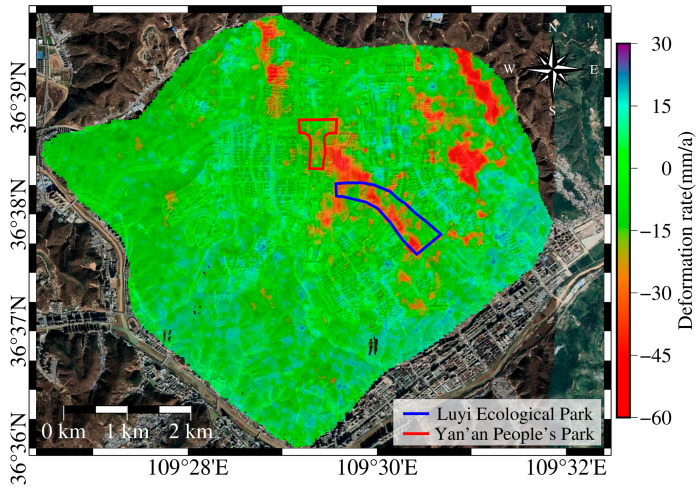
Distribution of Buildings in the Subsidence Area. Red and blue solid lines in the figure indicate the distribution of buildings constructed within the main subsidence areas of YND.

To ensure the safety of residents in the new area, construction should be avoided in regions with significant subsidence. According to the “Distribution Map of Key Construction Projects in the New Area North Zone” released by the YND Management Committee on 27 July 2015, the Lu Yi Ecological Park and Yan’an People’s Park are planned to be built in the Qiaoergou area, both located in regions with high subsidence rates. In other areas of notable subsidence, there are only a few buildings, primarily road facilities. Figure 17 shows that there is a significant spatial heterogeneity in subsidence rates within YND, with areas of higher subsidence typically having larger fill thickness, loose soil structures, and frequent fluctuations in groundwater levels. Therefore, it is essential to focus on these areas during urban planning to avoid high-rise buildings and heavy infrastructure. In response to the Lu Yi Ecological Park and Yan’an People’s Park, local authorities have taken measures to construct low-rise buildings in these areas and to build road facilities to reduce the risk of subsidence. This indicates that optimizing land use and building types can effectively mitigate potential geological disaster risks.

Areas with lower subsidence and uplift demonstrate relative stability, making them well-suited for high-rise buildings, public facilities, and commercial developments. High-rise structures require strong foundation load-bearing capacity, which is better supported in areas with low subsidence. Additionally, placing public facilities in stable zones reduces maintenance needs and extends their operational lifespan. Stable foundation conditions minimize subsidence risks for buildings, ensuring structural safety over time. Consequently, zones with minimal deformation present favorable foundational conditions for durable construction of buildings and infrastructure.

## 6. Conclusions

The land relocation and urban development plan in the YND is of strategic importance to the city of Yan’an. However, this initiative has significantly altered the original geological structure, leading to varying degrees of land subsidence across the district. In this study, the SBAS-InSAR technique is applied to Sentinel-1A imagery to investigate the spatial characteristics of ground deformation in YND following the substantial completion of construction activities in 2017. Furthermore, the study provides a detailed analysis of the underlying causes of severe subsidence in specific areas. The main conclusions are summarized as follows:

1. Based on Sentinel-1A imagery from 2017 to 2022, SBAS-InSAR monitoring revealed a clear subsidence trend in filled valley regions, with a maximum cumulative subsidence of 400 mm and a maximum subsidence rate of −89 mm/year. In contrast, excavation zones located on original mountain ridges exhibited only minor uplift, with a maximum deformation rate of 25 mm/year.
2. Precipitation and groundwater storage are the primary natural factors contributing to subsidence. In areas with high precipitation (≥60 mm/month), the subsidence rate increases significantly. Precipitation alters surface water conditions, which then infiltrates the subsurface and affects groundwater storage, subsequently influencing ground deformation. A significant positive correlation was found between cumulative subsidence and groundwater storage with a two-month lag, with most correlation coefficients ranging between 0.4 and 0.8.
3. Fill thickness, as the dominant anthropogenic factor, shows a positive correlation with surface deformation. Greater fill thickness prolongs the primary consolidation stage, resulting in a longer period of rapid subsidence. After project completion, the Yan’an municipal government actively promoted greening efforts and avoided construction in high-risk areas (subsidence rate >−40 mm/year), instead developing public parks to reduce accident risks and improve the ecological environment of the new district.

## Data Availability

The European Space Agency for providing Sentinel-1 data (https://search.asf.alaska.edu/#/), the National Tibetan Plateau Data Center of China for providing the precipitation data (http://doi.org/10.5281/zenodo.3185722) and NASA for providing the GLDAS CLSM data (https://disc.gsfc.nasa.gov/), the COP DEM is a product of the European Union’s Copernicus Programme and the ESA, it is available from the Copernicus Data Space Ecosystem (https://dataspace.copernicus.eu/). ASTER GDEM is a product of METI and NASA; it is available from NASA’s Earthdata portal (https://earthdata.nasa.gov/). The ZY-3 satellite data used in this study were provided by the China Centre for Resources Satellite Data and Application (CRESDA) (https://data.cresda.cn). The authors gratefully acknowledge the ISCE project (https://github.com/isce-framework/isce2), funded by the NASA-ISRO SAR (NISAR) project, and the time-series InSAR processing software MintPy (https://github.com/insarlab/MintPy), both of which provided essential support for data processing and analysis in this study.
